# Effect of limb rotation on radiographic alignment measurement in mal-aligned knees

**DOI:** 10.1186/s12938-021-00956-7

**Published:** 2021-11-27

**Authors:** Xiaoshu Sun, Bin Yang, Shengzhao Xiao, Yichen Yan, Zifan Liu, Liang Yuan, Xiaohua Wang, Bin Sun, Jie Yao, Yubo Fan

**Affiliations:** 1 Present Address: Key Laboratory of Biomechanics and Mechanobiology (Beihang University), Ministry of Education, Beijing Advanced Innovation Center for Biomedical Engineering, School of Biological Science and Medical Engineering, Beihang University, Beijing, People’s Republic of China; 2 Orthopedics Department, Peking University International Hospital, Life Park 1, Zhongguancun Life Science Park, Changping District, Beijing, People’s Republic of China; 3 Present Address: School of Medical Science and Engineering, Beihang University, No.37 Xueyuan Road, Haidian District, Beijing, 100083 People’s Republic of China

**Keywords:** Alignment measurement, Long-leg radiography, Knee flexion, Knee coronal deformity

## Abstract

**Purpose:**

Long-leg-radiography (LLR) is commonly used for the measurement of lower limb alignment. However, limb rotations during radiography may interfere with the alignment measurement. This study examines the effect of limb rotation on the accuracy of measurements based on the mechanical and anatomical axes of the femur and tibia, with variations in knee flexion and coronal deformity.

**Methods:**

Forty-five lower limbs of 30 patients were scanned with CT. Virtual LLRs simulating five rotational positions (neutral, ± 10$^{\circ }$, and ± 20$^{\circ }$ internal rotation) were generated from the CT images. Changes in the hip–knee–ankle angle (HKA) and the femorotibial angle (FTA) were measured on each image with respect to neutral values. These changes were related to knee flexion and coronal deformity under both weight- and non-weight-bearing conditions.

**Results:**

The measurement errors of the HKA and FTA derived from limb rotation were up to 4.84 ± 0.66$^{\circ }$ and 7.35 ± 0.88$^{\circ }$, respectively, and were correlated with knee flexion (*p* < 0.001) and severe coronal deformity (*p* < 0.001). Compared with the non-weight-bearing position, the coronal deformity measured in the weight-bearing condition was 2.62$^{\circ }$ greater, the correlation coefficients between the coronal deformity and the deviation ranges of HKA and FTA were also greater.

**Conclusions:**

Flexion and severe coronal deformity have a significant influence on the measurement error of lower limb alignment. Errors can be amplified in the weight-bearing condition compared with the non-weight-bearing condition. When using HKA and FTA to represent the mechanical axis and the anatomical axis on LLR, limb rotation impacts the anatomic axis more than the mechanical axis in patients with severe deformities. Considering LLR as the gold standard image modality, attention should be paid to the measurement of knee alignment. Especially for the possible errors derived from weight-bearing long-leg radiographs of patients with severe knee deformities.

**Supplementary Information:**

The online version contains supplementary material available at 10.1186/s12938-021-00956-7.

## Introduction

The lower limb alignment is an important reference for clinical diagnosis and treatment of conditions affecting the knee. Malalignment of the lower extremity is recognized as a critical component of the etiology of knee osteoarthritis [4, 17, 19]. Correct alignment is critical for the outcome of total knee arthroplasty (TKA) [12], especially for personalized surgical design [16, 21]. Lower limb alignment is commonly assessed using measurements of anatomical or mechanical (weight-bearing) axes of the femur and the tibia, as depicted on plain radiographs. Quantification of these axes usually requires the identification of anatomical landmarks from hip to ankle. For evaluation of the knee alignment, long-leg radiographs (LLR) are considered as the gold standard image modality, with high inter- and intra-observer reliability.

Correct and standardized patient positioning during imaging is the prerequisite for the accuracy of the lower limb alignment measurement while using LLR. However, considering patients with knee osteoarthritis or other joint disease are not able to extend the knee fully, it is reasonable to study the possible alignment measurement errors of patients with severe deformity. Studies have reported that limb rotation or foot rotation could significantly affect the outcome [6, 9, 11] [15]. The measurement error of hip–knee–ankle (HKA) angle could reach 4–10 degrees or even greater [14, 15]. In comparison, the recommended HKA after TKA ranges from − 3$^{\circ }$ to 3$^{\circ }$, which represents a proper knee alignment and prosthesis position. Therefore, the preoperative measurement error directly affects knee osteotomy. Previous studies have suggested that improved methods of measuring lower limb alignment are of greater importance to the advancement of knee osteotomy than the technical details of the surgical procedure itself [11, 13, 15].

Although studies involving synthetic prosthesis [11, 14], specimen of human lower limbs [3, 20] and in vivo images [7] all existed, the magnitude of these errors varies dramatically. Some researchers believed the long-leg radiograph is a reliable method [1, 10] and reported that the error caused by limb rotation was less than 1$^{\circ }$ within the acceptable range [18], whereas others found significant errors in the measurements of severe varus knees [8, 14, 15, 18]. Coronal deformity, knee flexion contracture, or some other anatomical features in patients might be important factors that potentially affect the magnitude of measurement errors. Considering the in vivo images derived from patients can better reflect real clinical application scenarios, the measurement error of knee alignment in patients with complex deformities should be investigated more in-depth. It is meaningful to quantify the magnitude of alignment measurement errors in patients with different degrees of deformity.

**Fig. 1:**
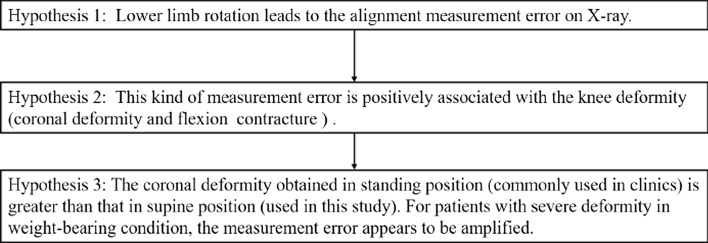
This study attempts to prove these hypotheses

The purposes of this study are: (1) to determine the influence of limb rotation on measurements of knee alignment derived from long-leg radiographs; (2) to measure the additional influence of coronal deformity and knee flexion contracture; and (3) to determine whether weight-bearing affects the accuracy of the radiographic measurements. The significance of this study is to assist in reducing measurement errors of mechanical and anatomical leg axis. This study attempts to prove the following hypotheses (Fig. 1).

## Results

**Fig. 2:**
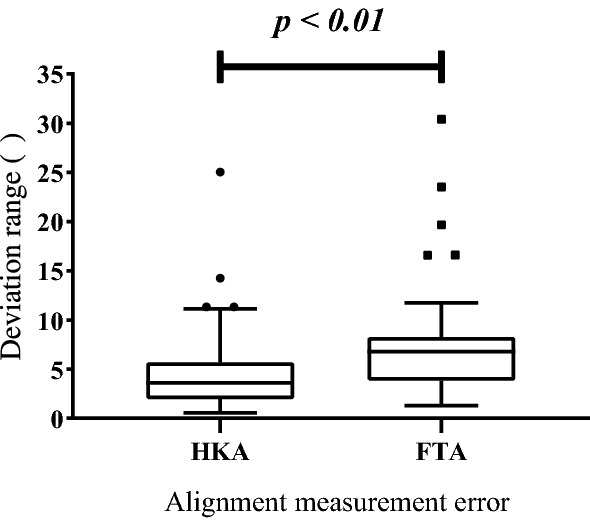
Box-plot illustrated the average deviation ranges of HKA and FTA. *p* value < 0.01, the difference between HKA and FTA deviation range was significant

The median value of knee flexion was 5.32$^{\circ }$ (2.27$^{\circ }$; 8.38 $^{\circ }$). The median knee coronal deformity was 7.37 $^{\circ }$ (4.25$^{\circ }$; 9.50$^{\circ }$). Descriptive statistics of the HKA and FTA deviation range are summarized in Table 1. The average deviation ranges of HKA and FTA were 4.84 ± 0.66$^{\circ }$ (range: 0.56–25.03) and 7.35 ± 0.88$^{\circ }$ (range: 1.30–30.42), respectively. As shown in Fig. 2, the mean difference between HKA and FTA deviation range was 2.50 (*p* < 0.01). The intraclass correlation coefficients for HKA and FTA deviation range were perfect, 0.976 (*p* < 0.05) and 0.935 (*p* < 0.05), respectively.

**Table 1:** HKA and FTA deviation range derived from varying degree of flexion and coronal deformity

	HKA deviation range	FTA deviation range
	(M ± S.E.M.)	(M ± S.E.M.)
Slight flexion group	2.15 ± 0.26$^{\circ }$	3.95 ± 0.45$^{\circ }$
Moderate flexion group	5.32 ± 0.39$^{\circ }$	7.47 ± 0.43$^{\circ }$
Severe flexion group	12.81 ± 2.20$^{\circ }$	18.19 ± 2.72$^{\circ }$
Slight coronal deformity group	4.57 ± 1.67$^{\circ }$	6.28 ± 1.95$^{\circ }$
Moderate coronal deformity group	4.43 ± 0.64$^{\circ }$	6.85 ± 0.87$^{\circ }$
Severe coronal deformity group	7.24 ± 1.29$^{\circ }$	11.92 ± 2.34$^{\circ }$
Total	4.84 ± 0.66$^{\circ }$	7.35 ± 0.88$^{\circ }$

Descriptive statistics

*M* mean, *SEM* standard error of mean

**Fig. 3:**
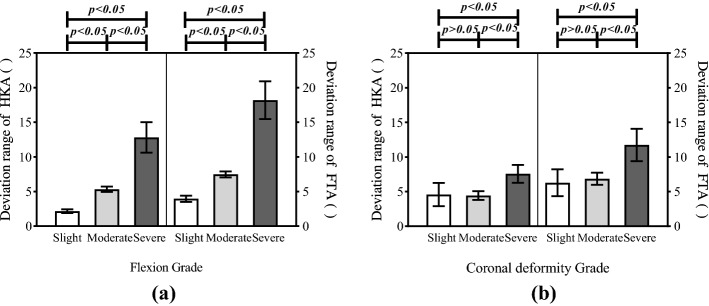
The deviation range of HKA and FTA in patients with different flexion and coronal deformity. *p* value < 0.05 means that difference between the two corresponding groups is significant in 95$\%$ confidence level. **a** Participants were divided into slight, moderate, and severe flexion groups by the degree of flexion contracture. The left part showed the HKA deviation range in the slight, moderate and severe flexion group. The right part showed the FTA deviation range in the slight, moderate and severe flexion group. **b** Participants were divided into slight, moderate, and severe coronal deformity groups by the degree of coronal deformity. The left part showed the HKA deviation range in the slight, moderate, and severe coronal deformity group. The right part showed the FTA deviation range in the slight, moderate and severe coronal deformity group

According to the two-way ANOVA: there were statistical differences in both HKA deviation range and FTA deviation range significantly for patients with varying flexion and coronal deformity (*p* < 0.05); the interaction between flexion and coronal deformity also had a significant effect on HKA and FTA deviation range (*p* < 0.05). Fig. 3 illustrates the average HKA and FTA deviation range in patients with different flexion and coronal deformities.

For flexion index, there were significant differences among the slight, moderate and severe groups in both HKA and FTA deviation range (*p* < 0.001) (Table 2). For the coronal deformity index, there was no significant difference between the slight and moderate groups in both HKA and FTA deviation range (*p* > 0.05). Yet, the coronal deformity index of the severe group was significantly different from that of the slight and moderate groups (*p* < 0.001) (Table 3). Among the different degrees of coronal deformity, only the severe group differed dramatically from the other two groups in the HKA deviation range (*p* < 0.001) and in the FTA deviation range (*p* < 0.001). No significant difference was found between the slight coronal deformity group and the moderate coronal deformity group in the HKA deviation range (*p* > 0.05) and in the FTA deviation range (*p* > 0.05).

**Table 2:** Multiple comparisons of flexion group, the mean difference of HKA deviation range and the mean difference of FTA deviation range

Flexion group	HKA deviation				FTA deviation			
	Mean difference	S.E	Sig.	95% C.I.	Mean difference	S.E	Sig.	95% C.I.
Slight–moderate	− 3.17*	0.49	< 0.001	− 4.16 $\sim$ − 2.17	− 3.51*	0.80	< 0.001	− 5.14 $\sim$− 1.89
Moderate–severe	− 8.04*	0.73	< 0.001	− 9.51 $\sim$ − 6.57	− 11.98*	1.18	< 0.001	− 14.38$\sim$− 9.59
Slight–severe	− 11.20*	0.70	< 0.001	− 12.63 $\sim$ − 9.78	− 15.50*	1.14	< 0.001	− 17.82 $\sim$− 13.18

*S.E* standard error, *Sig.* significance probability, *C.I.* confidence interval

*The mean difference is significant at the 0.05 level

**Table 3:** Multiple comparisons of coronal deformity group, the mean difference of HKA deviation range and the mean difference of FTA deviation range

Coronal deformity group	HKA deviation				FTA deviation			
	Mean difference	S.E	Sig.	95% C.I.	Mean difference	S.E	Sig.	95% C.I.
Slight–moderate	0.14	0.51	0.783	− 0.89$\sim$1.17	− 0.57	0.83	0.493	-2.26$\sim$1.11
Moderate–severe	− 2.81*	0.69	< 0.001	− 4.22$\sim$ − 1.40	− 5.06*	1.13	< 0.001	-7.35$\sim$-2.78
Slight–severe	− 2.67*	0.75	< 0.001	− 4.18$\sim$− 1.16	− 5.64*	1.21	< 0.001	− 8.10$\sim$− 3.18

*S.E* standard error, *Sig.* significance probability, *C.I.* confidence interval

*The mean difference is significant at the 0.05 level

**Table 4:** Correlation coefficients between the knee deformity and alignment measurement errors

Correlation coefficient	Knee flexion (non-weight-bearing)	Coronal deformity (non-weight-bearing)	Coronal deformity (weight-bearing)
Deviation range of HKA	0.933**	0.307*	0.635**
Deviation range of FTA	0.861**	0.504**	0.639**

The deviation range of HKA and FTA are specific parameters representing the measurement error of mechanical and anatomical alignment, respectively. The correlation coefficient greater than 0 indicates a positive correlation

*Correlation is significant at 0.05 level (two-tailed)

**Correlation is significant at 0.01 level (two-tailed)

The deviation ranges of HKA and FTA were significantly correlated with the knee flexion and coronal deformity (Table 4). In the non-weight-bearing condition, the correlation coefficient between coronal deformity and HKA deviation range was 0.307 (*p* < 0.05); the correlation coefficient between coronal deformity and with FTA deviation range was 0.504 (*p* < 0.01). In the weight-bearing condition, the correlation coefficient between coronal deformity and HKA deviation range was 0.635 (*p* < 0.01); the correlation coefficient between coronal deformity and with FTA deviation range was 0.639 (*p* < 0.01). Remarkable correlations were found between the flexion angle and deviation ranges of both HKA (0.933, *p* < 0.01) and FTA (0.861, *p* < 0.01).

**Fig. 4:**
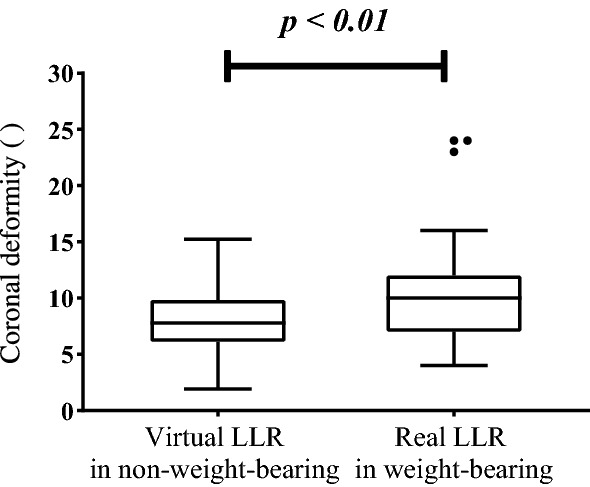
Box-plot illustrating the coronal deformity measured in neutral position. Virtual LLR: virtual radiographs simulated from full-leg CT scan in non-weight-bearing condition. Real LLR: standing full-leg radiographs in weight-bearing condition

The box-plot in Fig. 4 illustrates the coronal deformity measured from virtual LLR and real LLR. In the neutral position, coronal deformity measured on real LLR 10.52 ± 0.94$^{\circ }$was significantly greater than that on virtual LLR 7.90±0.54$^{\circ }$. The mean difference of coronal deformity in the non-weight-bearing condition and weight-bearing condition was 2.62 ± 0.68$^{\circ }$ (*p* < 0.01).

## Discussion

The lower limb alignment measurement is a crucial factor in the preoperative plan and postoperative assessment of a knee replacement. Inconsistent positioning during the capture of X-ray and resulting projected image can bring measurement errors of the lower limb alignment. However, the argument over whether these measurement errors are acceptable and related to the knee deformity has not been settled. This study quantitatively revealed that the influence of limb rotation on measurements of knee alignment derived from long-leg radiographs, the measurement errors corresponding to different deformed knees, and the difference of radiographic measurements on weight-bearing and non-weight-bearing conditions.

In this study, limb rotation caused the deviation ranges of HKA and FTA measurement to be greater than 3$^{\circ }$. The degree of the anatomical axis variation was greater than that of the mechanical axis. Although tilted projections can lead to measurement errors of the lower limb alignment, the magnitude of these errors measured in different studies varies dramatically. Wright JG [20] reported that the deviation range of FTA measurement was less than 1$^{\circ }$ under the limb rotation from 20$^{\circ }$ external rotation to 20$^{\circ }$ internal rotation, whereas Swanson KE [18] found significant errors in severe deformity knees up to 7.8$^{\circ }$. This could be explained by the fact that the degree of deformity of the lower limb would influence the measurement error of the alignment axis.

Our results demonstrated that the coronal deformity affected the alignment measurement errors greatly. Multiple comparisons of slight, moderate, and severe degrees of coronal deformity indicated that the severe group (coronal deformity > 10$^{\circ }$) differed significantly from the other two groups in alignment measurement errors. Compared with the non-weight-bearing condition, the coronal deformity of the weight-bearing condition had a greater influence on the measurement errors. However, the effects of coronal deformity on the assessment of alignment on LLR in the previous studies were controversial. Swanson et al. [18] discussed in saw-bones research of alignment measurements in deformed limbs that the FTA measurement in limbs with severe valgus or varus deformity was more sensitive to the effect of rotation than in normally aligned limbs.This is in line with our results that mechanical and anatomical axis measurement errors in the severe coronal deformity group were greater than those in the slight or moderate group. Jud et al. [7] conclude that deviations in mechanical leg axis measurements did not vary relevantly through the coronal deformity. It is an interesting conclusion that appears to contradict the findings of our study. Still, it is, in fact, compatible with this study’s conclusions because they only analyzed coronary malformations below 9 degrees. The coronal deformities of our research subjects ranged from 0.62 to 19.55 degrees. No significant difference was found between the slight and the moderate coronal deformity group in alignment measurement errors. While the severe group (coronal deformity > 10$^{\circ }$) had significant differences with the other two groups in alignment measurement errors. Additionally, this study measured both the HKA and FTA deviation range of each subject. On the contrary, the study conducted by Kawakami et al. [8] reported no significant correlation between coronal deformity and measurement error. Kawakami et al. set different limb rotation ranges for different individuals to calculate the measurement error. In the present study, the limb rotation from internal 20$^{\circ }$ to external 20$^{\circ }$ was performed uniformly for each subject. And the present study further collected coronal deformity in the weight-bearing condition to compare with the result obtained from the non-weight-bearing condition. The opposite conclusion was probably reached from these two aspects. Therefore, the hypothesis that coronal deformity and the measurement errors were significantly related is credible. The effect of coronal deformity on the assessment of alignment on real LLR may be more pronounced. The method of alignment measurement should vary from slight to severe patients of varus and valgus.

The knee flexion angle plays an important role in the potential error of the alignment measurement. Multiple comparisons of the slight, moderate and severe flexion group showed significant differences in alignment measurement error for all combinations. With the increase of knee flexion, the measurement error of lower limb alignment increased significantly. According to a study with a synthetic model by Lonner et al. [11], the FTA deviation range of samples with flexion varied more than the control group. This statement was consistent with our study, and a similar conclusion was reached in a radiographic cadaver study conducted by Brouwer et al. [3]. Differently, the present study recruited clinical patients instead of using synthetic models or specimens of human lower limbs. Both knee osteoarthritis and normal individuals under clinical conditions were observed. Since the bony anatomy varies widely, the lower limbs alignment in vivo state can reflect the real morphology information. On the other hand, the knee flexion of each subject was not preset by the researchers but appeared naturally. Knee flexion angles were calculated by the self-developed program for quantitative analysis. The difference in the measured coronal deformity was significant between the weight-bearing condition and the non-weight-bearing condition. Brouwer et al. [2] performed a standing and a supine LLR in 20 patients with varus deformity and found an average of 2$^{\circ }$ more varus deviation in the standing position than in the supine position. In our study, the virtual LLR reconstructed from CT scanning in the supine position was used to compare with the real LLR in the standing position. The average HKA measured on the virtual LLR is 2.83$^{\circ }$ less than that on the real LLR. This could be explained by the reduction of loading forces across the knee joint in the supine position. Although the clinical examination of standing LLR is different from the supine position LLR we used, it is not expected to alter the trends of the above results observed from the virtual LLR in the present study.

There are some limitations to this study. Firstly, the virtual LLRs were simulated from supine CT scanning, which resulted in a slight difference from the clinical examinations. It is preferable to obtain the CT images in the weight-bearing condition. Consequently, this study compared the lower limb alignment measured on virtual LLRs with the real LLRs to complement our results obtained in the non-weight-bearing position. Secondly, when exploring the relationship between knee coronal deformity and measurement errors in the weight-bearing condition, it is not rigorous enough to use HKA reflected on the real LLR to represent knee coronal deformity. However, this provides some clues to the relation between coronal deformity and measurement errors in clinics. Thirdly, the gender-specific differences of coronal alignment in osteoarthritic knees were not clear according to a review by Hess et al. [5]. The study participants were primarily females (73$\%$), and thus, we could not show a gender difference. Some studies proposed that assessing the lower limb alignment in subjects with an uneven gender ratio might be biased. The last limitation is associated with the low proportion of severe lower limb malformation subjects. However, current results demonstrated a trend that the more severe the deformity, the greater the measurement error of lower limb alignment.

## Conclusions

The alignment measurement of the lower limb on LLR can be affected by the limb rotation. However, the measurement errors caused by this effect vary individually. This kind of measurement error is positively associated with the knee deformity (coronal deformity and flexion contracture). When comparing the varying coronal deformity group, the severe group (coronal deformity>10$^{\circ }$) differed significantly from the other two groups in the HKA deviation range and in the FTA deviation range. Moreover, compared with the non-weight-bearing condition, the influence of coronal deformity on measurement errors will be amplified in the weight-bearing condition. In addition, the error of measuring the anatomic axis on LLR is greater than that of the mechanical axis. Therefore, attention should be paid to the errors of measuring lower limb alignment on LLR, especially for patients with severe knee deformity in the weight-bearing condition.

## Materials and methods

### Subjects and imaging procedure

**Table 5:** Patient baseline demographics and image information collection in clinics

	Participants (*n* = 30)
Median age, years (median 25th; 75th quartiles)	67.0 (62.0; 67.5)
Female, *n* ($\%$)	22 (73.3$\%$)
Bilateral knee, *n* ($\%$)	15 (50$\%$)
Non-weight-bearing CT scan, *n* ($\%$)	30 (100$\%$)
Weight-bearing LLR image, *n* ($\%$)	21 (70$\%$)

From May 2018 to November 2019, 30 patients (45 knees) with a median age of 67 (range from 48 to 94) were enrolled in this study (Table 5). Twenty-two patients (33 knees) were female, and eight patients (12 knees) were males. Patients with a history of bone loss, infection, tumor, congenital disease, and lower extremity surgery were excluded from participation. CT scans of all subjects (45 knees) and weight-bearing LLR for some patients (31 Knees) with knee arthritis were detected preoperatively. Radiographs were performed with 7–22 mAs and 70–90 kVp, depending on the body mass. CT scans (Siemens SOMATOM Definition Flash, Germany) of the lower extremities in the supine position were obtained using a 1.0 mm section thickness with 120KVp. The method of measuring the knee flexion and coronal deformity is shown in Supplementary Materials. The knee flexion in the supine position and coronal deformity of each patient appeared naturally were calculated by a custom program. According to the degree of knee flexion and coronal deformity in the non-weight-bearing condition, 45 knees were graded as shown in Table 6. The degree of knee flexion and coronal deformity is divided by three levels: 0–5 degrees, 5–10 degrees, more than 10 degrees.

**Table 6:** According to the degree of knee flexion and coronal deformity, 45 knees were classified into the following levels

Grade	Flexion or coronal deformity angle	Knee (*N* = 45)
Slight flexion	0–5$^{\circ }$	22 (48.9$\%$)
Moderate flexion	5–10$^{\circ }$	17 (37.8$\%$)
Severe flexion	> 10$^{\circ }$	6 (13.3$\%$)
Slight coronal deformity	0–5$^{\circ }$	14 (31.1$\%$)
Moderate coronal deformity	5–10$^{\circ }$	25 (55.6$\%$)
Severe coronal deformity	> 10$^{\circ }$	6 (13.3$\%$)

### Alignment measurement

**Fig. 5:**
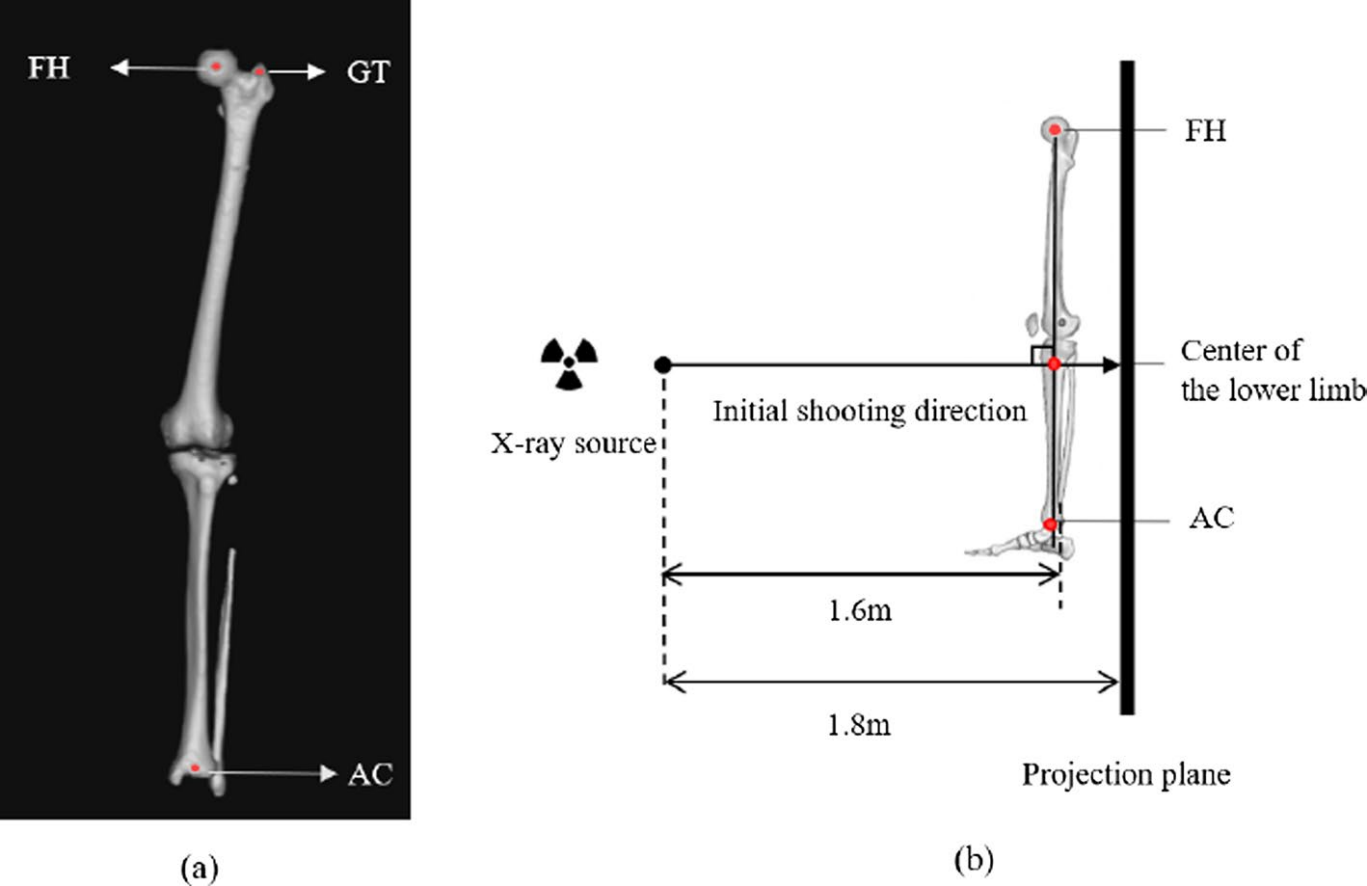
Applying the 3D model of the lower limb reconstructed from CT scanning to set parameters and geometric relations for virtual X-ray capturing. **a** Three landmarks to determine anatomical planes on the 3D model: the center of the femoral head (FH), the center of ankle joint (AC), and the tip of the greater trochanter (GT). **b** The schematic diagram of capturing virtual X-rays by using the lower limb 3D model. The center of the lower limb was defined as the midpoint of line FH–AC. The X-ray source was 1.6 and 1.8 m away from the center of the lower limb and the projection plane, respectively. The initial shooting direction was perpendicular to line FH–AC, passing through the center of the lower limb

The determination of anatomical planes depends on three landmarks from 3D models (Fig. 5a): center of the femoral head (FH), the center of ankle joint (AC), and the tip of the greater trochanter (GT). Specifically, FH was defined as the well-fitting sphere center point of the femoral head; AC was defined as the diagonal intersection of the ankle joint surface; GT was defined as the tip of the greater trochanter from the frontal forward direction. Then the coronal plane was defined as the plane passing through Points FH, AC, and GT. The sagittal plane was defined as the plane perpendicular to the coronal plane and passing through line FH–AC.

**Fig. 6:**
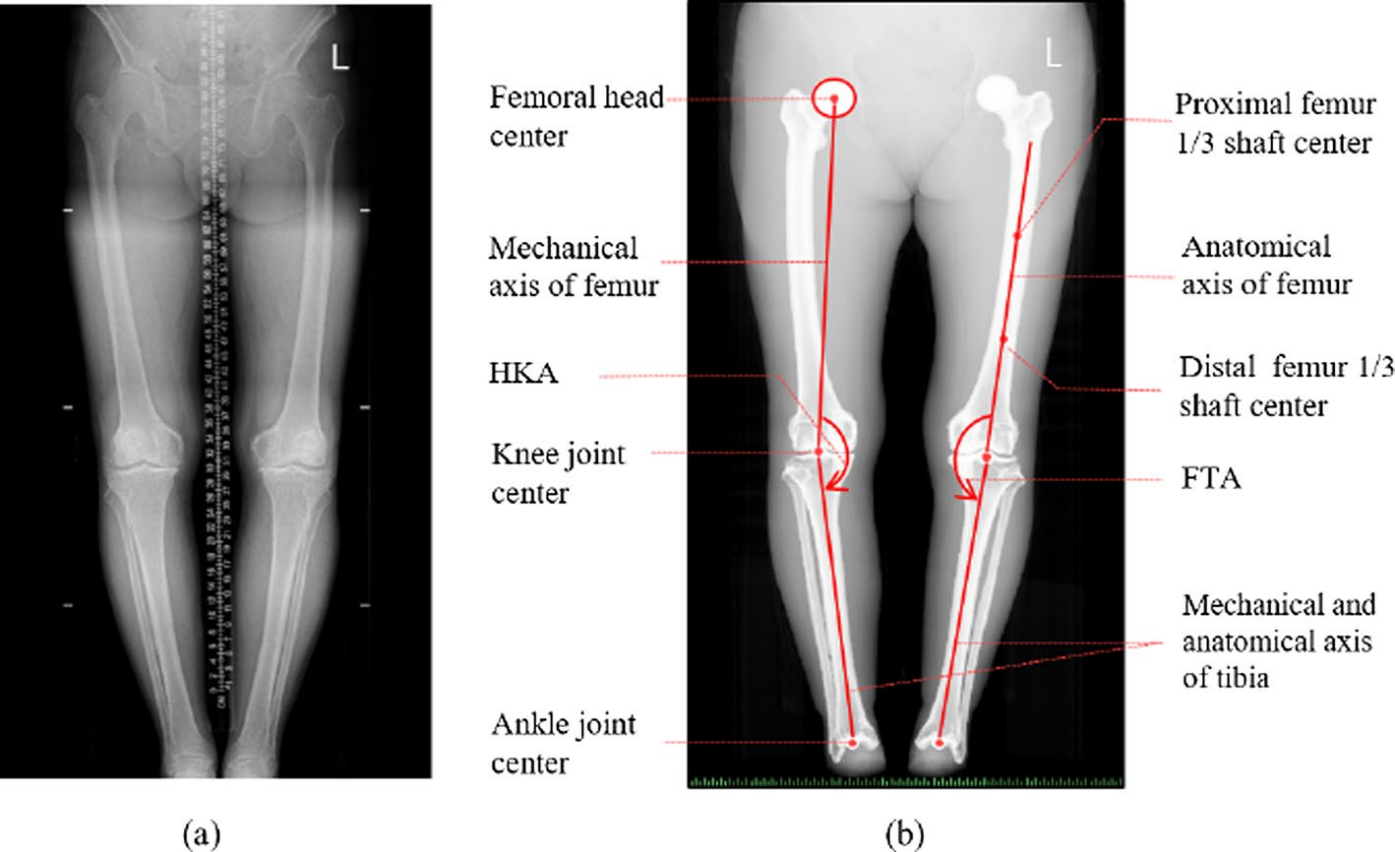
A real long-leg radiograph in clinics and a virtual long-leg radiograph in this study. **a** The real long-leg radiograph was captured in weight-bearing condition. **b** The virtual long-leg radiograph was captured in the non-weight-bearing condition. Both mechanical axis and anatomical axis were measured on virtual LLR. The HKA was defined as the mechanical axial of the femur and tibia. The FTA was defined as the anatomical axial of the femur and tibia

To measure the axial alignment of non-weight-bearing conditions and compare them with weight-bearing LLRs, the virtual LLRs were reconstructed by a Python program. The X-ray source was positioned at 1.6 meters forward from the midpoint of line FH-AC, and the projection plane was 1.8 meters from the X-ray source. The initial beam direction was oriented perpendicular to the coronal plane. Figure 5b shows the schematic of capturing virtual LLR. The virtual LLR of each knee was reconstructed from the projection of the 3D model by using a self-developed python program. Real LLR in the weight-bearing condition and virtual LLR in the non-weight-bearing condition are shown in Fig. 6. From the radiograph, the mechanical axis of the femur was defined as the line passing from the center of the femoral head to the center of the knee joint; the anatomical axis of the femur was defined as the line passing midpoint of the upper and lower 1/3 of the femoral medullary cavity; both the mechanical and anatomical axis of the tibia was defined as the line passing the center of the knee and ankle joint. HKA was defined as the medial side angle of the mechanical axis; FTA was defined as the medial side angle of the anatomical axis. Both HKA and FTA were measured on virtual LLR, as shown in Fig. 6a.

To simulate the clinical, radiographic capture, and quantify the variability of HKAs and FTAs measured on LLR, the 3D model was rotated from the neutral position. The rotation axis was defined as the line FH–AC. With the rotation of the lower limb in 10$^{\circ }$ increments, ranging from 20$^{\circ }$ internally to 20$^{\circ }$ externally, five series of virtual LLRs were obtained. The range (maximum minus minimum) of HKAs and FTAs in each group was defined as the HKA deviation and the FTA deviation, respectively.

### Statistical analysis

To assess the impact of knee coronal deformity and flexion angle on the deviation of HKA and FTA, two-way ANOVAs were calculated using SPSS 22.0 (SPSS Corp, Chicago, USA). The threshold for statistical significance was set at alpha = 0.05. The normality test and uniformity test of error variance are carried out for the raw data. Following that, multiple comparison least significant difference (LSD) was run to compare the mean changes between each event. In addition, Spearman’s correlation coefficients were accessed to analyze the relationship between variables. The method for performing the knee flexion and coronal deformity measurement was shown in Supplementary Materials. The percentages for categorical variables were summarized. Means, standard deviations, and the standard error of mean or medians with interquartile ranges were performed for continuous variables. The differences of coronal deformity between virtual LLR (non-weight-bearing condition) and real LLR (weight-bearing condition) were analyzed by a paired *t*-test. Intra-observer reliability was calculated by the intraclass correlation coefficient (ICC). The interpretation scales of ICC are classified according to the following: 0–0.20 is slight, 0.21–0.40 is fair, 0.41–0.60 is moderate, 0.61–0.80 is substantial, and 0.81–1.00 is perfect.

## Supplementary Information

**Additional file 1.** Method for performing knee flexion and coronal deformity measurement.

## Data Availability

The datasets during and/or analyzed during the current study are available from the corresponding author on reasonable request.

## Abbreviations

LLR: Long-leg-radiography
HKA: Hip–knee–ankle angle
FTA: Femorotibial angle
TKA: Total knee arthroplasty
ANOVA: Analysis of variance
FH: Femoral head
AC: Center of ankle
GT: Greater trochanter
LSD: Least significant difference
ICC: Intraclass correlation coefficient
