# Supplementary material for: Effect of limb rotation on radiographic alignment measurement in mal-aligned knees
Source: Biomed Eng Online. 2021 Nov 27;20:119. doi: 10.1186/s12938-021-00956-7 (PMC8626971; doi:10.1186/s12938-021-00956-7)
Supplement: Supplementary file 1 — Additional file 1. Method for performing knee flexion and coronal deformity measurement. [file 12938_2021_956_MOESM1_ESM.pdf]

Supplementary Materials

The method of measuring the knee coronal and flexion angle

Non-weight-bearing condition:

Coronal deformities (degree of knee varus) and flexion angles (degree of flexion contracture) were defined by spatial angles based on the three-dimensional solid models reconstructed from CT images rather than the clinically used projection angles between the femoral and tibial mechanical axis on two-dimensional plane which is commonly used in clinics. This is because most of the motions occurring in anatomical joint are three-dimensional motions involving 6 degrees of freedom, including three translations and three rotations. So, using the projection angles, the measurement of coronal deformities and flexion angles might interfere with each other. For example, the projection angle of mechanical axis obtained at 0° flexion are different from that obtained at other flexion angles. Extremely, no matter how great the varus deformity is, the projection angle of the coronal deformity measured in the 90° flexion condition is always 90°. Considering that CT images in the supine position were taken in this study, in non-weight bearing condition, the method for measuring lower extremity deformities in space was defined as the angle between mechanical axis and quasi-sagittal plan or quasi-coronal plane. The details of method defining the knee flexion and coronal deformity in three-dimension are as follow.

To measure the knee varus and flexion angle in non-weight-bearing condition, the quasi-sagittal and quasi-coronal planes were arranged to describe the spatial angles. In addition to FH, GT, and AC, the position of the center of the knee (KC) on the 3D model should also be determined (Fig.S1). KC was defined as the midpoint of the well-fitting circle of the tibial plateau. The quasi-coronal plane of femur was defined as the plane passing through FH, GT, and KC. The quasi-sagittal plane was perpendicular to the quasi-coronal plane through FH and KC. The tibial mechanical axis on 3D model was defined as line KC-AC. Knee coronal deformity was defined as the angle between the mechanical axis of the tibia and quasi-sagittal plane of femur. The knee flexion angle was defined as the angle between the tibial mechanical axis and quasi-coronal plane.

Weight-bearing condition:

The measurement of knee coronal deformity in weight-bearing condition was conducted on the real LLR. Knee coronal deformity was defined as the angle between the mechanical axis of femur and tibia in two-dimension.

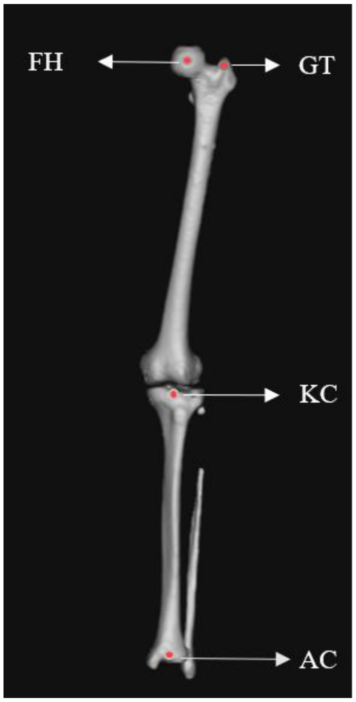

Fig.S1 Four landmarks to determine mechanical and anatomical axis on 3D model: center of the femoral head (FH), center of ankle joint (AC), and tip of the greater trochanter (GT).

Original Data

	Knee Flexion degree（non-weight-bearing）	Coronal Deformity degree(non-weight-bearing)	Coronal Deformity degree (weight-bearing)	HKA in -20° rotation	HKA in -10° rotation	HKA in 0° rotation	HKA in 10° rotation	HKA in 20° rotation
subject1	7.67	12.36	24.00	170.76	168.66	166.48	165.50	163.68
subject2	6.54	7.43	16.00	175.55	173.74	171.92	170.62	169.95
subject3	4.59	9.83	10.00	172.89	172.15	170.94	170.49	169.44
subject4	7.09	12.39	12.00	170.46	171.06	168.55	168.04	168.38
subject5	6.83	10.72	13.00	171.78	170.76	169.51	169.30	168.90
subject6	0.96	4.37	4.00	177.08	176.52	176.72	176.69	176.66
subject7	7.37	7.37	/	175.99	174.67	173.63	172.26	171.31
subject8	0.88	7.12	4.00	174.50	173.70	173.54	173.90	173.61
subject9	8.47	8.75	7.76	176.19	174.53	173.12	172.18	171.70
subject10	4.32	8.80	10.00	173.56	172.63	171.91	171.86	172.16
subject11	6.75	9.92	12.00	173.67	171.98	171.15	170.03	169.07
subject12	2.29	7.24	10.00	174.59	174.41	174.37	173.62	173.31
subject13	9.28	11.92	13.00	173.17	170.45	168.27	166.77	164.80
subject14	0.60	9.08	10.00	172.08	171.60	171.43	171.49	171.42
subject15	0.14	6.80	8.00	173.38	173.56	172.95	173.24	173.25
subject16	3.75	10.71	11.00	172.35	170.70	169.17	168.72	167.59
subject17	2.85	6.12	6.00	175.90	175.12	174.51	174.63	173.31
subject18	4.39	7.78	10.00	175.10	173.93	172.75	172.44	171.75
subject19	9.88	3.25	7.00	180.05	177.97	176.14	174.10	172.36
subject20	4.29	4.82	8.00	177.69	176.89	175.81	174.83	174.25
subject21	6.15	8.41	13.00	173.45	171.90	171.10	169.45	168.99
subject22	7.41	8.76	12.00	174.21	172.48	171.07	169.81	169.57
subject23	15.09	15.23	24.00	170.58	167.13	163.90	161.43	159.26
subject24	0.35	1.74	/	177.78	176.88	177.14	176.91	177.37
subject25	0.26	2.10	/	178.04	178.46	178.31	178.68	178.74
subject26	8.75	0.95	/	176.39	177.89	179.61	181.31	182.17
subject27	7.30	1.84	/	176.14	177.92	178.86	180.02	181.49
subject28	2.33	3.55	/	185.97	185.43	184.11	183.63	182.74
subject29	3.64	11.28	/	193.32	193.22	191.49	190.77	190.26
subject30	0.61	3.40	6.00	176.86	177.52	177.16	177.56	177.73
subject31	0.90	6.74	8.00	186.71	187.62	187.97	187.84	187.62
subject32	2.73	5.84	/	183.88	184.99	186.21	186.85	187.40
subject33	1.93	6.15	6.50	184.51	185.19	185.75	186.46	186.60
subject34	10.01	5.67	8.50	180.54	183.20	184.90	186.63	188.20
subject35	18.36	7.73	23.00	194.97	194.55	192.08	189.91	187.30
subject36	1.90	4.13	5.00	179.75	180.01	180.24	180.26	180.58
subject37	36.95	0.63	/	193.01	186.88	181.19	174.20	167.99
subject38	23.13	8.02	/	194.98	192.25	188.45	184.65	180.72
subject39	16.53	15.27	/	171.40	167.65	164.61	162.87	160.06
subject40	15.32	19.55	/	201.74	201.21	199.59	197.72	194.75
subject41	2.23	4.72	/	184.85	184.93	185.03	184.85	184.66
subject42	4.84	0.62	/	181.73	181.01	180.40	179.58	178.35
subject43	8.05	8.11	10.74	179.65	179.19	179.75	179.25	179.39
subject44	8.07	9.16	8.54	179.45	179.37	179.55	179.53	179.22
subject45	5.48	1.92	5.00	184.22	183.03	182.81	181.77	181.20
